# The Role of *Malassezia* spp. in Atopic Dermatitis

**DOI:** 10.3390/jcm4061217

**Published:** 2015-05-29

**Authors:** Martin Glatz, Philipp P. Bosshard, Wolfram Hoetzenecker, Peter Schmid-Grendelmeier

**Affiliations:** Allergy Unit, Department of Dermatology, University Hospital of Zurich, Gloriastrasse 31, 8091 Zurich, Switzerland; E-Mails: philipp.bosshard@usz.ch (P.P.B.); Wolfram.Hoetzenecker@usz.ch (W.H.)

**Keywords:** atopic dermatitis, *Malassezia* spp., IgE antibodies, cytokines, auto-reactive T cells

## Abstract

*Malassezia* spp. is a genus of lipophilic yeasts and comprises the most common fungi on healthy human skin. Despite its role as a commensal on healthy human skin, *Malassezia* spp. is attributed a pathogenic role in atopic dermatitis. The mechanisms by which *Malassezia* spp. may contribute to the pathogenesis of atopic dermatitis are not fully understood. Here, we review the latest findings on the pathogenetic role of *Malassezia* spp. in atopic dermatitis (AD). For example, *Malassezia* spp. produces a variety of immunogenic proteins that elicit the production of specific IgE antibodies and may induce the release of pro-inflammatory cytokines. In addition, *Malassezia* spp. induces auto-reactive T cells that cross-react between fungal proteins and their human counterparts. These mechanisms contribute to skin inflammation in atopic dermatitis and therefore influence the course of this disorder. Finally, we discuss the possible benefit of an anti-*Malassezia* spp. treatment in patients with atopic dermatitis.

## 1. Introduction

Atopic dermatitis (AD) is a frequent chronic relapsing inflammatory skin disorder. It is characterized by intensely itchy skin eczema and is frequently associated with allergic rhino-conjunctivitis and allergic asthma. The prevalence of AD in industrialized countries has tripled during the past 30 years [1], affecting 15%–30% of children and up to 10% of adults [2]. Despite its frequency and impact on public health, we do not fully understand the pathogenesis of AD. It appears to be in parts genetically determined and several factors seem to contribute to the development of AD. For example, the skin of AD patients is characterized by an impaired skin barrier function with increased trans-epidermal water loss, increased surface pH, reduced stratum corneum hydration and reduced expression of tight junction components [3]. Second, the skin immune system in AD patients is altered compared to healthy individuals [4]. For example, AD patients have increased levels of the cytokines interleukin (IL-) 4, IL-10 and IL-13 in their skin compared to healthy controls. These cytokines reduce the production of the antimicrobial peptides LL-37, human beta defensin (hBD)-2, and hBD-3 in keratinocytes [5,6,7], which are important components of the skin’s innate immune system in the defense against microorganisms. It can be speculated that the impaired skin barrier function and the altered skin immune system may play intertwining roles and contribute to colonization and growth of microorganisms on the skin of AD patients [3,7]. The altered skin colonization with microorganisms in AD patients *versus* healthy individuals has been extensively investigated for bacteria, in particular *Staphylococcus aureus*. During recent years, AD research has also focused on the possible pathogenetic correlation between eczema and the skin commensal fungus *Malassezia* spp. because AD patients are often sensitized to *Malassezia* spp.; and AD patients may benefit from an antifungal therapy that is effective against *Malassezia* spp. This led to the publication of a plethora of studies on the possible role of *Malassezia* spp. in the development and course of AD. Here, we will review the biology of *Malassezia* spp. on human skin and the current state of research on the role of *Malassezia* spp. in AD.

## 2. *Malassezia* spp. As Part of the Normal and Atopic Skin Flora

**Figure 1:**
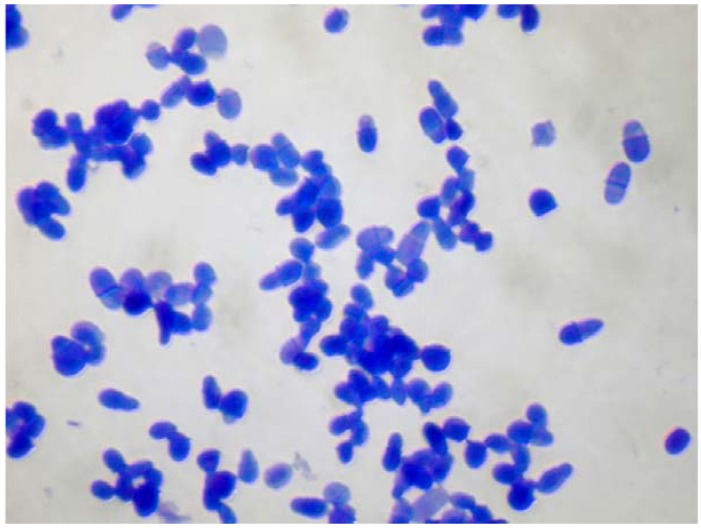
Microscopic image of a *Malassezia* spp. culture. Methylene blue staining.

The skin is an ecosystem and harbors diverse and body site-specific microbial communities, which have been termed the skin microbiome. The phylogenetic profiling of the skin microbiome revealed that fungi are part of the normal skin flora at all body sites and comprise 1%–22% of the phylogenetic composition of the skin microbiome [8]. The fungal flora of the healthy skin almost exclusively comprises *Malassezia* spp., and *Malassezia* spp. is therefore the main eukaryotic member of the microbial flora of the skin [8,9]. *Malassezia* spp. is a genus of lipophilic yeasts (Figure 1). Most of the species within this genus lack the genes for fatty acid synthase genes and therefore rely on exogenous fatty acid sources to satisfy their nutritive requirement [10]. *M. pachydermatis*, a species isolated from dogs and other animals [11], is the only known *Malassezia* species that grows in the absence of exogenous lipids [10]. Their need for exogenous lipids explains the predilection of *Malassezia* species for seborrheic skin sites, such as the head and neck.

The taxonomy of *Malassezia* spp. has been controversial since its recognition as a member of the human skin flora in the mid-19th century. The taxonomy was defined in its current form in 1996, based on morphology, ultrastructure, physiology and molecular biology [12]. *Malassezia* spp. belongs to the phylum Basidiomycota and currently encompasses 14 species that have been isolated from human and animal skin. Two of these species, *M. globosa* and *M. restricta*, predominate on human skin and are identified in almost all individuals and body sites [9,13]. Several studies investigated the epidemiology of *Malassezia* spp. in healthy and diseased skin by culture and molecular methods such as polymerase chain reaction [14,15,16,17,18,19,20,21,22,23]. These studies obtained variable results presumably owing to methodical inconsistencies between the studies; for example, the sampling sites were inconsistent between these studies. Next generation sequencing revealed that the skin fungal microbiome is highly site specific between body sites [13]. Therefore, comparing the prevalence of *Malassezia* species between different body sites sampled in different studies will give unreliable results. In addition, the epidemiological studies used different culture media to detect *Malassezia* species, and it was shown that different culture media favor the growth of particular *Malassezia* species [24,25].Therefore, the use of only one or a few types of culture media does not necessarily depict the whole spectrum of *Malassezia* species present in a sample. Despite these methodical considerations, epidemiological studies indicated a geographical variation in the distribution of particular *Malassezia* species, presumably owing to climate factors. For example, *M. sympodialis* has been reported in studies from Canada, Russia and Sweden as the most frequent species, whereas in Japan *M. furfur* was the most common species [14,15,16,17,18,19,20,21,22,23]. Of importance, studies comparing healthy individuals and AD patients did not reveal a difference in the frequency of skin colonization with *Malassezia* spp. between both groups [14,15,16,17,18,19,20,21,22,23].

## 3. Sensitization to *Malassezia* spp. May Correlate with the Severity of Atopic Dermatitis

*Malassezia* spp. as part of the healthy skin flora regularly interacts with the skin immune system. Therefore *Malassezia* spp.-specific IgG and IgM antibodies can be detected in healthy individuals [10]. However, healthy individuals are usually not sensitized to *Malassezia* spp., while a high proportion of AD patients is sensitized to this yeast [26]. This was demonstrated by positive atopy patch tests, skin prick tests or the presence of *Malassezia* spp.-specific IgE antibodies [26]. For example, 30%–80% of adult AD patients have a positive skin prick test with *Malassezia* spp. extract [27,28,29,30]. Unfortunately, standardized skin test extracts for *Malassezia* spp. are not yet commercially available, making it difficult to compare the results of different studies. In contrast, *Malassezia* spp.-specific serum IgE can be measured using a commercial and standardized kit (ImmunoCAP^®^ m70, Phadia) based on *M. sympodialis* (ATCC strain 42,132). Recently, a new kit containing several species of *Malassezia* has been introduced (ImmunoCAP^®^ m227) with a slightly increased sensitivity compared to the single species test according to our experience [31]. Using these commercial kits, *Malassezia* spp.-specific IgE are found in 5%–27% of children [27,32,33,34] and 29%–65% of adults with AD [27,31,34,35,36], which is consistent with the rates found by skin prick tests. Sensitization rates against *Malassezia* spp. are particularly higher in patients with head and neck types of AD [31]. Therefore, some authors assume that *Malassezia* spp. plays a pathogenetic role, particularly in this type of AD [37].

**Figure 2:**
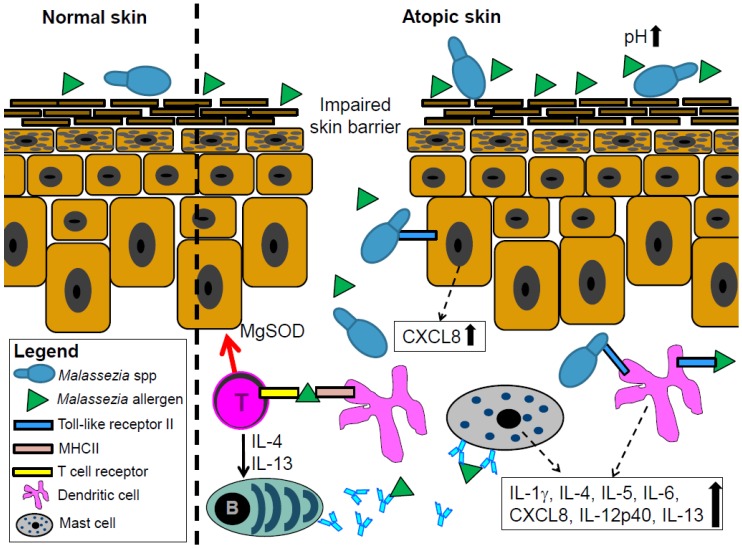
Proposed mechanisms by which *Malassezia* spp. contributes to skin inflammation in atopic dermatitis (AD) patients. The increased pH in atopic skin contributes to increased allergen release by *Malassezia* spp. These allergens, supposedly together with whole *Malassezia* spp. cells, penetrate the epidermis through the disturbed skin barrier in AD patients. *Malassezia* spp. cells and their allergens may be recognized by toll-like receptor 2 expressed on keratinocytes and dendritic cells which elicits the release of pro-inflammatory cytokines. *Malassezia* spp. components elicit the production of *Malassezia* spp.-specific IgE antibodies through the dendritic cells and T cell-mediated activation of B cells. These IgE antibodies may also contribute, possibly through mast cells, to the inflammation in atopic skin. Finally, autoreactive T cells can cross react between fungal and human manganese-dependent superoxide dismutase (MgSOD) and hence sustain skin inflammation.

The reason for the high frequency of *Malassezia* spp.-sensitization in AD patients compared to healthy individuals is still unclear but is attributed to a combination of dysfunctional skin barrier, genetic background, and environmental factors [38]. Several recent studies investigated a possible correlation between AD severity and the IgE-mediated sensitization to *Malassezia* spp. Our group analyzed 132 children and 67 adults with AD and found a significant correlation between the severity of AD and sensitization to *Malassezia* spp.-specific IgE in adults but not in children [34]. These results substantiated previous findings in 61 adult AD patients from Japan [39]. The lower frequency of *Malassezia* spp. sensitization in children compared to adults and the missing correlation between AD severity and *Malassezia* spp.-specific IgE in children might owe to the poor growth conditions for *Malassezia* spp. in children compared to adults. The lipid content of sebum, which is a prerequisite for skin colonization with most *Malassezia* spp. is low in children but rises during puberty and is high until the age of 50 [40]. Accordingly, sensitization to *Malassezia* spp. seems to occur preferably in adulthood, while sensitization to food allergens and aeroallergens frequently occurs during childhood [34]. Several allergens of *Malassezia* spp. elucidate a specific IgE response (Figure 2). To date, 13 allergens from two *Malassezia* species, *M. furfur* and *M. sympodialis*, are listed in the official allergen nomenclature list (http://www.allergen.org). In vitro experiments confirmed that *Malassezia* spp. release more allergens in the less acidic environment of pH 6.0 that represents conditions of atopic skin, than in the more acidic environment of pH 5.5 of healthy skin [41] (Figure 2). However, despite the frequent sensitization of adult AD patients against *Malassezia* spp., it is unclear if the IgE response plays a pathogenetic role in AD or rather serves as a marker for the severity of AD.

## 4. *Malassezia* spp. Interacts with the Skin Immune System

The interaction between *Malassezia* spp. and the skin immune system supposedly contributes to skin inflammation in AD patients. For example, the *Malassezia* spp. allergen Mala s 13 is a fungal thioredoxin that is very similar to its human homolog. CD4^+^ T cells that react against the fungal thioredoxin of *Malassezia* spp. are fully cross-reactive to the human enzyme. Therefore, besides recognizing the fungal enzyme, these T cells cross-react with the human enzyme, what may contribute to skin inflammation in AD [42]. A similar induction of autoreactive T cells was observed for a different *Malassezia* spp. allergen; Mala s 11 is a manganese-dependent superoxide dismutase with a high degree of sequence identity to the corresponding human enzyme. Similar to Mala s 13, Mala s 11 activates T cells that then react against the human homolog and sustain skin inflammation (Figure 2). These findings were substantiated by the strong correlation between AD severity and sensitization to Mala s 11 [43]. A protein from *M. globosa*, MGL_1304, was recently identified in the sweat of AD patients and is a potential allergen that may contribute to skin inflammation in AD [44]. The sensitization to this protein also positively correlates with the severity of AD [45].

Prior studies indicated that *Malassezia* spp. cells interact with various types of human skin and immune cells. This induces a pro-inflammatory immune response by the skin and immune cells, which may maintain skin inflammation in AD. It is still unclear how this interaction between *Malassezia* spp. cells and host cells occurs, and at least two possible ways of interaction are hypothesized. First, the impaired skin barrier facilitates the physical encounter between *Malassezia* spp. cells and cells of the epidermis and dermis, such as keratinocytes, Langerhans cells, dermal dendritic cells, natural killer cells and fibroblasts [46]. A second possible mechanism of *Malassezia* spp.—human cell interaction—might be mediated by immunogenic proteins of *Malassezia* spp. These proteins can be released in nanovesicles [47] and it was demonstrated that these proteins that might be present in these nanovesicles induce an increased release of TNF-alpha, IL-6, IL-8, IL-10 and IL-12p70 by dendritic cells and mast cells [48,49]. Other proteins such as MGL_1304 induce the degranulation of mast cells and the release of IL-4 by basophils [44].

Some authors suggest that Toll-like receptors (TLRs) mediate the communication between whole *Malassezia* spp. cells or their immunogenic proteins and human cells. TLRs are members of the large family of pattern recognition receptors, which play a key role in the innate immune system as they recognize molecules that are commonly shared by pathogens. It is known that particularly TLR2 recognizes components of yeast such as *Malassezia* spp. [7]. Some recent findings substantiated the relevance of TLRs for the immune response of human cells against *Malassezia* spp. For example, *Malassezia* spp. induces the expression of TLR2 and TLR4 on human keratinocytes [50,51], which mediate the increased production of the antimicrobial peptide human beta defensin 2 and the chemokine CXLC8 [51]. Others attribute the pro-inflammatory response of dendritic cells against *Malassezia* spp. to TLR-mediated mechanisms [46] (Figure 2).

The interaction between *Malassezia* spp. cells and human cells elicits a cytokine release by the human cells [3,52,53]. For example, various *Malassezia* species activate the NLRP3 inflammasome in skin dendritic cells and induce the production of IL-1β, IL-4, IL-5, IL-13, and IL-18 [3,52,53]. Additionally, mast cells of AD patients release increased amounts of IL-6 in response to *M. sympodialis* exposure [54]. This cytokine release from human cells in response to *Malassezia* spp. is suggested to contribute to the skin inflammation in AD (Figure 2).

### Therapeutic Approaches with Antifungals in AD

The most effective therapy for AD patients comprises excellent skin care to reconstitute the impaired skin barrier, anti-inflammatory treatment most commonly with topical steroids or calcineurin inhibitors, and the identification and elimination of trigger factors [38]. The significance of an antifungal therapy for AD has been discussed since many years. Azole antifungals are the most common class of antifungal drugs prescribed for AD patients. *In vitro*, azole antifungals are effective against *Malassezia* spp. [55,56] but susceptibility testing of *M. pachydermatis*, a species most commonly isolated from dogs, showed that strains isolated from dogs with AD were less susceptible to azole antifungals than strains isolated from healthy dogs [57]. The relevance of this finding for *Malassezia* isolates from humans remains to be elucidated.

In a clinical routine, the topical application of ketoconazole on the face of patients with AD of the head and neck type frequently improves eczema, presumably due to a partial overlap with seborrhoic dermatitis in some cases. However, a placebo-controlled study with topical miconazole-hydrocortisone cream and ketoconazole shampoo in AD of the head and neck type did not show any difference to treatment with hydrocortisone alone [58]. The effect of topical antifungals alone in AD compared with topical steroids or calcineurin inhibitors has not yet been investigated to date. The benefit of a systemic antifungal treatment for AD patients has been assessed in several randomized, placebo-controlled trials. A study compared 36 AD patients treated with ketoconazole with 39 AD patients treated with placebo. AD severity improved significantly in the ketoconazole group but not in the placebo group [59]. In another trial, a total of 53 AD patients were treated with either two different dosages of itraconazole or placebo. The improvement of AD severity was significantly higher in itraconazole treated patients than in the placebo group [60]. These positive effects of antifungals were not confirmed by another study comparing 15 AD patients treated with ketoconazole with 14 AD patients treated with placebo. Notably, both treatment groups received topical steroids. Although AD severity improved in both treatment groups, this improvement was not correlated to ketoconazole but rather to the topical steroids [61]. The ambiguous results of these clinical trials might be attributed to a selection bias. It can be speculated that antifungal therapies are more effective in one subgroup of AD patients than in another, for example in patients with a head-neck type of eczema. More recently published studies were of less quality, for example they comprised retrospective observations and lacked a standardized scoring system to assess the severity of AD [62]. Such trials are not appropriate to finally decide on a positive benefit of antifungal therapy in AD patients. Therefore, more randomized, placebo-controlled studies to assess the benefit of antifungal therapy in AD are needed. These studies should be designed to determine the subgroup of AD patients that benefits most from an antifungal treatment and to determine the optimal treatment regimen.

Interestingly, azole antifungals such as ketoconazole or itraconazole have anti-inflammatory properties. It was shown that these drugs inhibit the production of IL-4 and IL-5 by T cells, which might reduce skin inflammation and therefore contribute to improvement of eczema during antifungal treatment [63].

Another therapeutic approach for AD patients specifically against *Malassezia* spp. might be photodynamic therapy. *M. furfur* was cultured in the presence of a cationic photosensitizer. The irradiation with a 670-nm diode laser significantly reduced the viablity of *M. furfur* culture, depending on irradiation duration and photosensitizer dosage [64]. However, the relevance of these results in clinical routine still needs to be proved.

## 5. Conclusions

In summary, there is little doubt that *Malassezia* spp. plays a role in AD. This yeast may interact with the skin immune system, which is facilitated by the impaired barrier function of atopic skin, and sensitization against *Malassezia* spp. correlates with the activity of AD. In addition, antifungal therapy shows beneficial effects in some patients. However, the pathogenetic mechanism and mutual interaction between *Malassezia* and AD still remain partly unclear and need further investigation.
